# Current practice in the management of acromioclavicular joint dislocations; a national survey in the Netherlands

**DOI:** 10.1007/s00068-020-01414-0

**Published:** 2020-06-13

**Authors:** Philippe P. De Rooij, Esther M. M. Van Lieshout, Ivo J. Schurink, Michael H. J. Verhofstad, ACJ injury study group

**Affiliations:** Trauma Research Unit Department of Surgery, Erasmus MC, University Medical Center Rotterdam, P.O. Box 2040, 3000 CA Rotterdam, The Netherlands

**Keywords:** Acromio-clavicular joint, ACJ injury, Rockwood, Shoulder, Survey, Trauma

## Abstract

**Purpose:**

The aim of this study was to investigate current practice in the management of acromioclavicular joint dislocations in the Netherlands.

**Methods:**

A 36-item literature-based and expert consensus survey was developed. If available, one orthopaedic and one trauma surgeon for every hospital (*n* = 82) in the Netherlands was asked to complete the online questionnaire. Only complete data sets were included in the analysis. Descriptive analysis was performed using SPSS.

**Results:**

Of 149 invited surgeons, 106 (71%) fully completed the survey. The diagnosis of ACJ injury was mainly based on physical examination (91%) and radiographs (95%). The vast majority of patients with ACJ injuries was treated non-operatively. The decision for operative treatment was mainly based on the surgeon’s experience and available literature. Patient-related factors that contributed most to the decision to operate or not, were mainly functional needs and age. Cosmesis and gender contributed less to this decision. Rockwood II and III ACJ injuries were usually treated non-operatively, whereas Rockwood IV and V ACJ injuries were usually treated operatively. For primary and secondary operative treatment, a flexible implant was preferred over rigid fixation techniques. All respondents agreed that nonoperative treatment of Rockwood II ACJ injuries leads to satisfactory results and that secondary operative treatment is only rarely required. Also the majority of patients with Rockwood III ACJ injuries is treated non-operatively, although failure rates are considered higher.

**Conclusion:**

This survey showed a significant individual variation on diagnosis and treatment strategies among surgeons in the Netherlands. The majority of the Dutch surgeons concern a flexible implant the best available technique for patients who require operative treatment.

**Electronic supplementary material:**

The online version of this article (10.1007/s00068-020-01414-0) contains supplementary material, which is available to authorized users.

## Background

Acromioclavicular joint (ACJ) dislocations are common injuries. Currently, the classification of ACJ dislocations according to Rockwood is most commonly used. The incidence ratio between partially ligamentous injuries (Rockwood I–II) and fully ligamentous injuries (Rockwood III–VI) is approximately 2:1 [1]. Fully ligamentous injuries appear to be unstable in anteroposterior direction at the physical examination of the shoulder.

There is consensus about nonoperative treatment of Rockwood type I and II injuries [2]. Concerning fully ligamentous injuries with severe dislocation (Rockwood type IV–VI) there is consensus that operative treatment is the best option [1]. Whether or not Rockwood type III injuries should be treated operatively remains subject to debate [3–7].

A difficulty in many published studies is that no standardized surgical techniques were compared. Besides autograft techniques such as of Weaver–Dunn and other autologous tendon reconstructions, a diversity of implants have been used: acromioclavicular k-wire fixation, Bosworth screw (rigid coracoclavicular fixation), clavicle hook plate, and different coracoclavicular suture techniques [5–7]. Many of these implants cause pain despite correct implantation. Other disadvantages concern secondary implant migration and the need for removal to achieve a normal range of motion. The majority of studies that compared these operative techniques with nonoperative treatment showed superior results for nonoperative treatment.

Four relatively recent reviews could still not conclude which treatment is the best for Rockwood III injuries [8–11]. The final conclusion of the Cochrane review was: “There is insufficient evidence from randomized controlled trials to determine when surgical treatment is indicated for acromioclavicular dislocation in adults in current practice. Sufficiently powered, good quality, well-reported randomized trials of currently-used surgical interventions versus conservative treatment for well-defined injuries are required.”

Based on the available literature, modern implants and fixation techniques, a clear direction on how to treat ACJ-dislocations is hard to point out. Because of the lack of clear scientific evidence, it is likely that the choice of treatment is dependent on the surgeon’s preference and/or local protocols. The aim of this national survey among (Orthopaedic) Trauma Surgeons was to investigate current practice in the management of acromioclavicular joint dislocations in the Netherlands.

## Methods

This questionnaire study was conducted and reported in accordance with the guidelines for survey research of Bennett et al. [12].

### Questionnaire

The questionnaire was drafted in Dutch and critically appraised by four trauma surgeons and an epidemiologist to ensure relevance, completeness, linguistics and style. The final version of the questionnaire consisted of a total of 36 questions; five for participant’s information, 27 questions related to (1) the number of patients treated; (2) the use of classification and diagnostic modalities; and (3) the treatment of Rockwood II, III, IV, and V ACJ dislocations. The final four questions were statements on the need for operative treatment in specific types of patients. Validity and reliability of the questionnaire were not assessed. The full questionnaire is available in English (Online Appendix 1).

### Selection of respondents

The departments of Trauma Surgery and General Orthopedic Surgery of each hospital in The Netherlands (*N* = 82) were asked to provide the name of the trauma surgeon and orthopedic surgeon with the most affinity with the topic of ACJ dislocations. If a hospital had multiple locations, only one location with the most trauma-related admissions was selected. All named surgeons were approached by telephone and informed about the aims and method of the survey. Respondents were asked to provide the information for their entire department. Since every hospital across the country was asked to participate, the targeted group of surgeons was presumed to be an adequate representation of the nationwide level of knowledge and care for patients with an ACJ injury. A sample size calculation was not necessary for this survey.

### Distribution of survey

The questionnaire was distributed online using LimeSurvey software (Version 2.05+, LimeSurvey Project Team, Carsten Schmitz (2015), LimeSurvey Project Hamburg, Germany). After obtaining verbal informed consent each surgeon received a link to the questionnaire and unique and secure access codes by email. The first invitation was sent on January 27, 2017. Reminders were sent every 2 weeks until the survey was closed on December 14, 2017.

### Data and statistical analysis

Data were stored online by a secured function of the software used. Upon closure of the survey, the data were downloaded to an SPSS file. Only complete data sets were included in the analysis. All completed questionnaires were analyzed in one group. Descriptive data analysis was done using SPSS version 22.0 (SPSS Statistics for Windows, Released 2012, Armonk, New York, IBM Corporation). All data were of categorical nature and are shown as numbers with corresponding percentages.

## Results

### Respondent characteristics

Of the 149 invited surgeons, 126 (85%) responded. Twenty provided a partial response and the other 106 (71%) fully completed the survey. The median age of the 106 respondents was 46 years (P_25_–P_75_ 42–53) and 100 (94%) were male. The respondents worked in a general hospital (*n* = 95; 90%) or University medical center (*n* = 11; 10%). Fifty-eight respondents (55%) had more than 10 years of experience as consultant orthopedic trauma surgeon (Table 1).

**Table 1:** Years of practice of the respondents as consultant orthopedic trauma surgeon

	N (%)
< 5 years	11 (10%)
5–9 years	37 (35%)
10–19 years	40 (38%)
20 years or longer	18 (17%)

Data are shown as *N* (%)

Fifty-five (52%) respondents treated annually between 20 and 50 patients with any ACJ injury. Only two respondents (2%) treated less than five patients each year (Table 2).

**Table 2:** Number of patients treated annually at the department for any acute ACJ injury

	*N* (%)
< 5 patients	2 (2%)
5–9 patients	5 (5%)
10–19 patients	29 (27%)
20–49 patients	55 (52%)
50 patients or more	15 (14%)

Data are shown as *N* (%) and include both operatively and non-operatively treated patients

### Diagnosis and classification

Sixty-five (61%) respondents used the Rockwood classification and 41 (39%) used the Tossy classification.

**Fig. 1:**
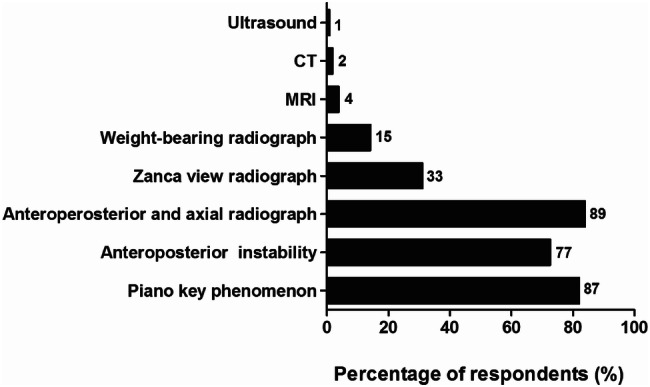
Diagnostics used for diagnosing ACJ injuries. The number of respondents is given next to the bars

The diagnosis of an ACJ injury was mainly based on a combination of physical examination and radiographs (Fig. 1). Ninety-six (91%) respondents assessed the piano key phenomenon or AP instability, and radiographs were used by 101 (95%) respondents. Of the five respondents who did not use X-ray, none used other radiodiagnostic tools like ultrasound, CT-scan, or MRI.

**Fig. 2:**
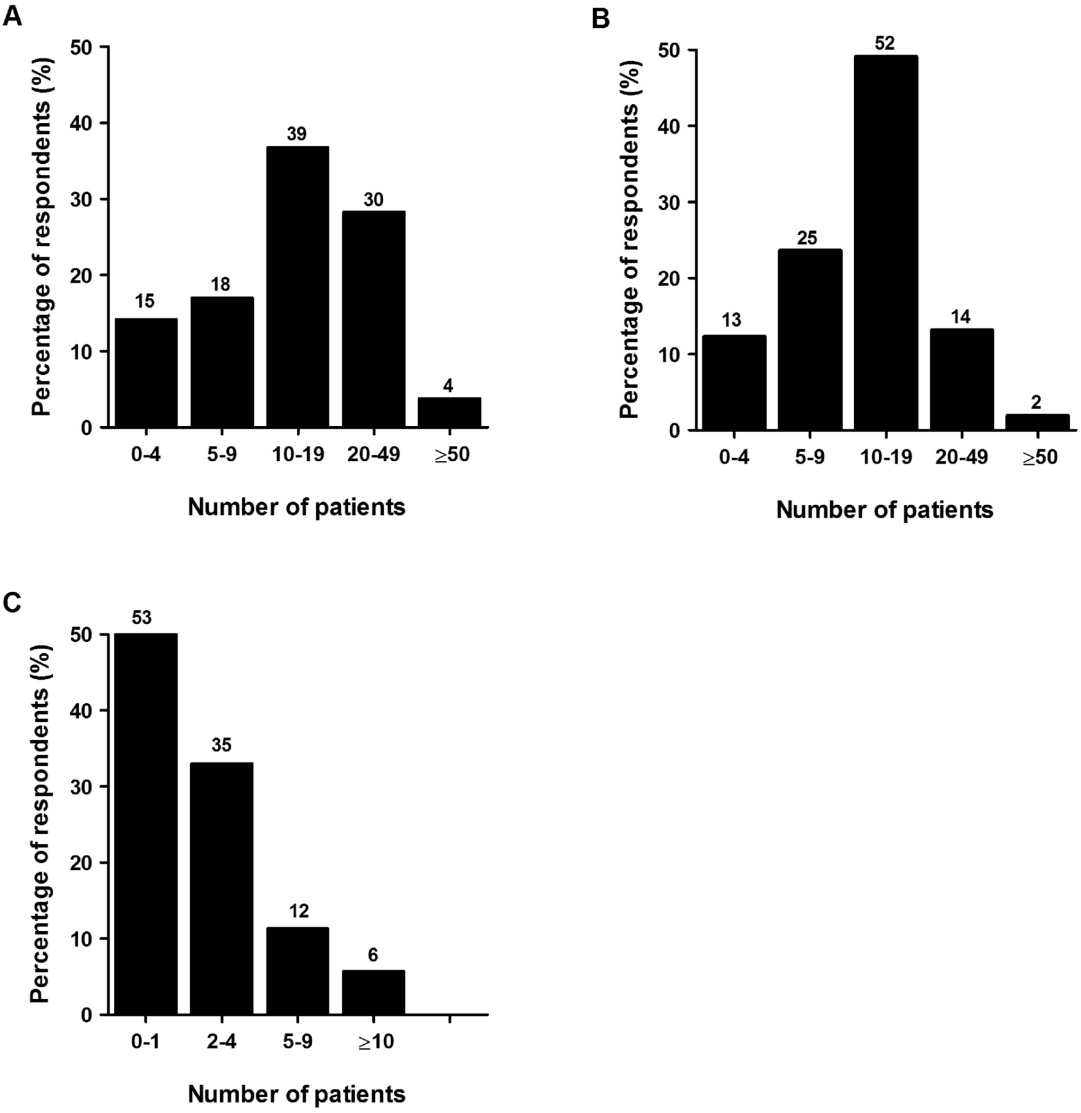
Number of patients annually treated for **a** Rockwood II, **b** Rockwood III, and **c** Rockwood V ACJ injuries. The number of respondents is given above the bars

The majority of the respondents stated to treat 10 to 50 patients with Rockwood II (*n* = 69; 65%), 5–19 patients with Rockwood III (*n* = 77; 73%), but only 0–4 patients with Rockwood V ACJ injuries per year (Fig. 2a–c).

### Treatment

Seventy (66%) respondents reported to have a treatment protocol for acute ACJ injuries in their hospital.

**Fig. 3:**
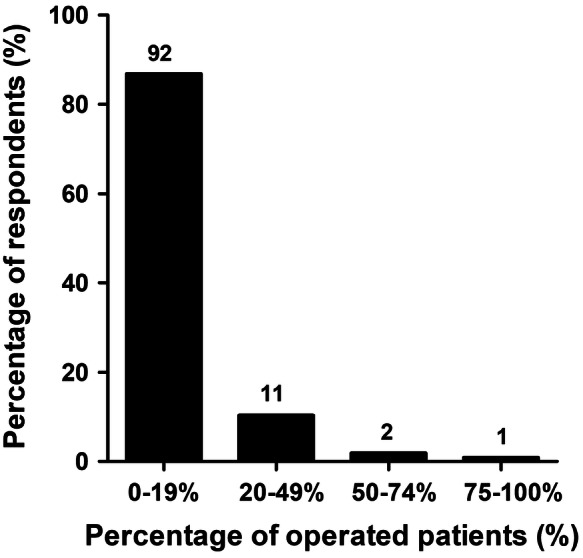
Percentage of patients with any ACJ injury treated operatively within 2 weeks after trauma. The number of respondents is given above the bars

**Fig. 4:**
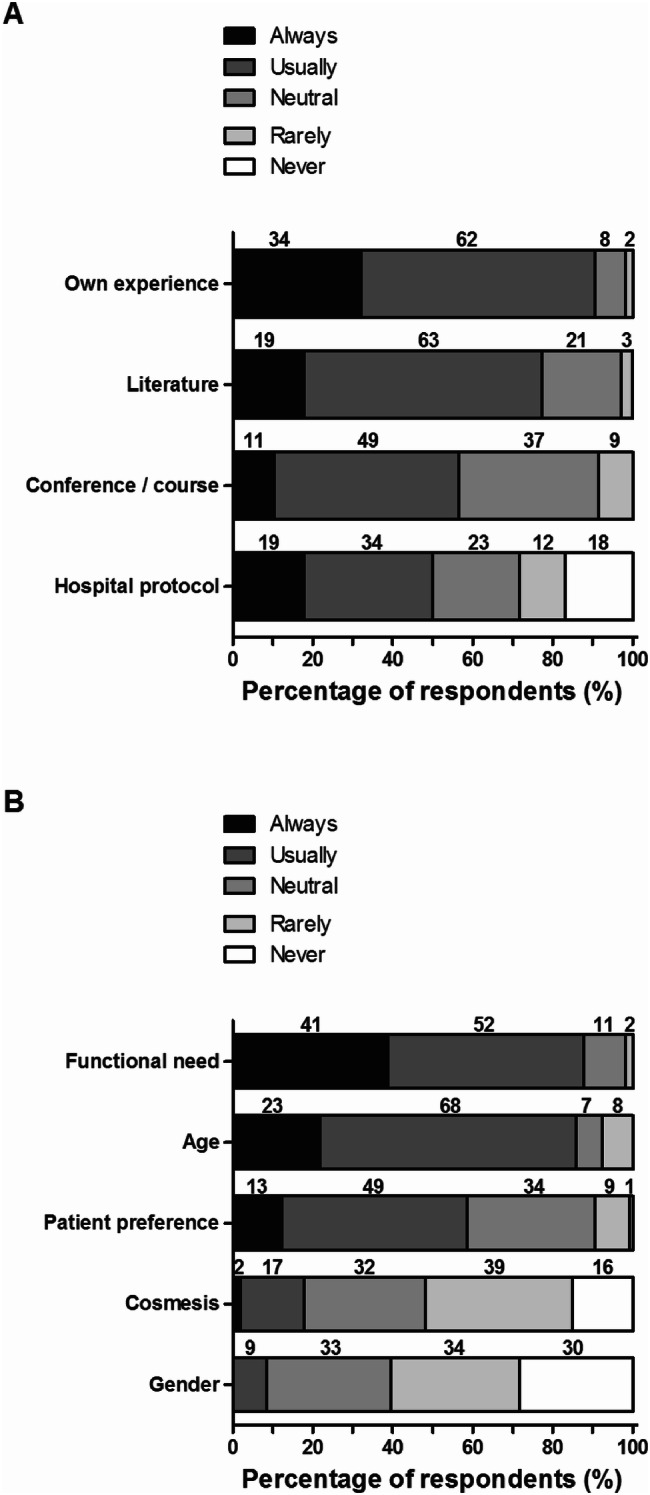
Physician-related (**a**) and patient-specific (**b**) factor contributing to the decision for operative treatment of any ACJ injuries. The number of respondents is given above the bars

The vast majority of ACJ injuries is treated nonoperatively in the primary setting (defined as within two weeks after injury; Fig. 3). A total of 92 respondents stated that they operated only 0–19% of patients with any type of ACJ injury. Figure 4 shows the factors that contributed to the decision for operative treatment of any ACJ injury; “usually” or “always” are indicated as a positive valuation. The most important physician-related factors were own experience (*n* = 96; 91%) and available literature (*n* = 82; 77%). The most important patient-related factors were functional need (*n* = 93; 88%) and age (*n* = 91; 86%). Cosmesis (18%) and gender (9%) contributed less to the treatment decision.

**Fig. 5:**
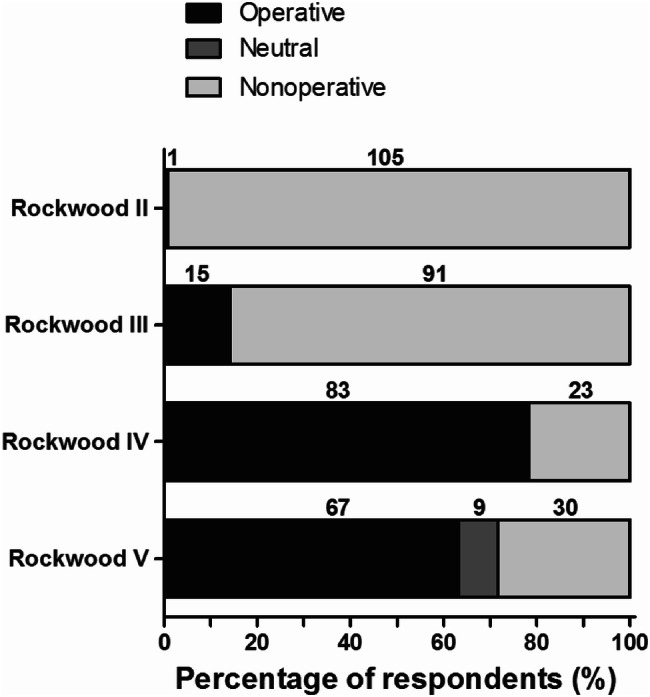
Type of preferred primary treatment for Rockwood II, III, IV, and V ACJ injuries. The number of respondents is given above the bars

Figure 5 shows the respondents preferred primary treatment for ACJ injuries. Nonoperative treatment was mostly preferred for Rockwood II (*n* = 105; 99%) and Rockwood III (*n* = 91; 86%) ACJ injuries. Operative treatment was preferred for Rockwood IV (*n* = 83; 78%) and Rockwood V (*n* = 67; 63%) ACJ injuries.

Fifty-seven (54%) respondents made a deliberate distinction between the diagnosis of Rockwood III and Rockwood V ACJ injuries, and 63 (59%) would treat a Rockwood III different than a Rockwood V ACJ injury. However, only nine (9%) reported to base this discrimination on additional radio-diagnostic tests, such as CT-scan (*n* = 5; 5%), weight-bearing radiograph (*n* = 4; 4%), MRI (*n* = 2; 2%) or ultrasound (*n* = 1; 1%).

**Fig. 6:**
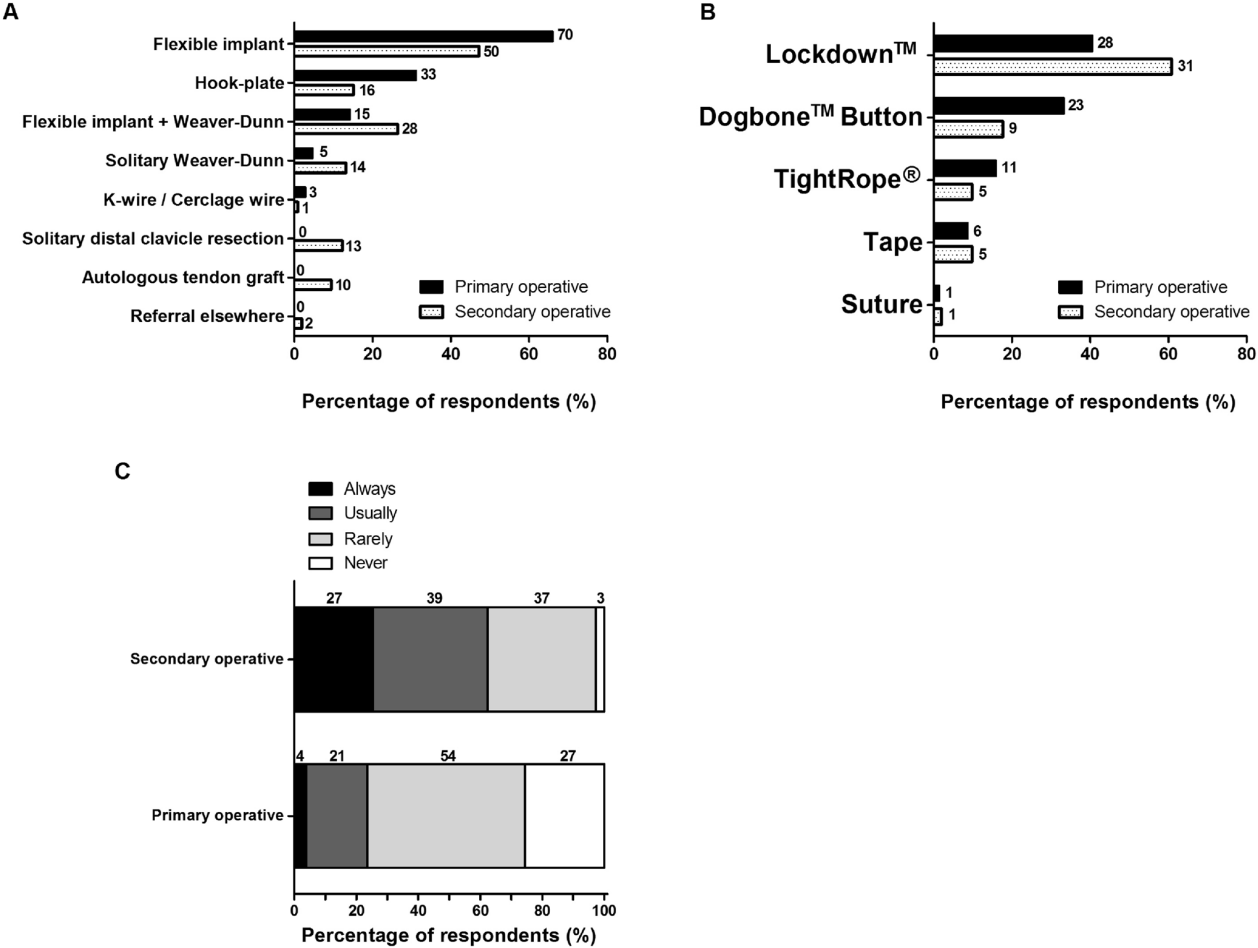
**a** Top 3 of treatment strategies, **b** flexible implants, and **c** need for distal clavicle resection for primary and secondary operative treatment strategies used for ACJ injuries. The number of respondents is given next to the bars

Figure 6a shows the respondents’ top three of primary and secondary operative treatment strategies used for any ACJ injury. Primary operative treatment was defined as the operative treatment of acute ACJ injury within two weeks after injury. Secondary operative treatment was defined as an operative treatment after failed nonoperative management of ACJ injuries. For primary operative treatment, 70 (66%) respondents used a “flexible implant”, 33 (31%) a clavicular hook plate or 15 (14%) a flexible implant combined with a Weaver–Dunn procedure. For secondary operative treatment, 50 (47%) respondents used a “flexible implant” only, 28 (26%) combined a flexible implant with a Weaver–Dunn procedure or 16 (15%) used a clavicular hook plate. Solitary distal clavicle resection (*n* = 13; 12%) or autologous tendon graft (*n* = 10; 9%) were secondary operative techniques, however, not mentioned as a primary operative technique. None of the respondents had selected the Bosworth coracoclavicular screw as a preferred treatment option, neither for primary nor secondary treatment.

Figure 6b shows the preferred types of flexible implants used. For primary as well as secondary operative treatment, the Lockdown™ was most frequently used.

In Fig. 6c, the need for distal clavicle resection is shown: 25 (24%) and 66 (62%) respondents “usually” or “always” performed a distal clavicle resection during primary or secondary operative treatment, respectively.

### Results of treatment

**Fig. 7:**
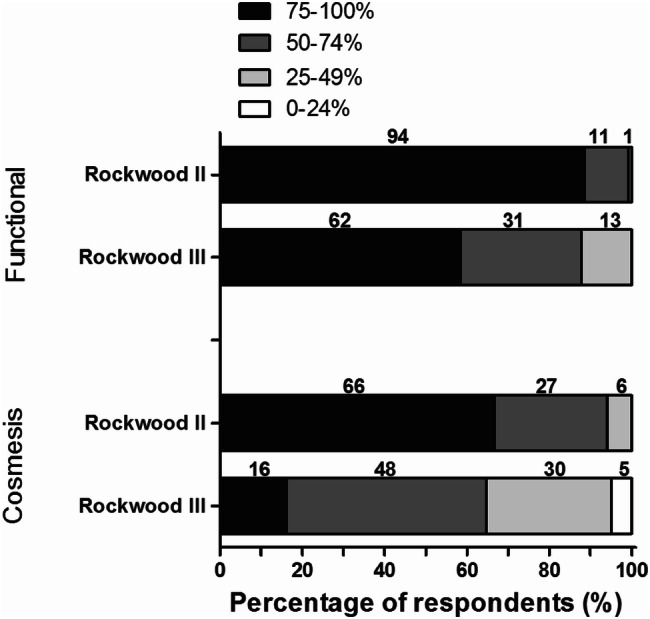
Respondents’ opinion on the proportion of patients that are satisfied with the functional and cosmetic results at 1 year after nonoperative treatment of ACJ injuries. For Rockwood II ACJ-injuries, seven respondents reported to be undecided. The number of respondents is given above the bars

Figure 7 shows the opinion of the respondents on the proportion of patients that are satisfied with the functional and cosmetic results at 1 year after nonoperative treatment of Rockwood II and III ACJ injuries. Concerning Rockwood II ACJ injuries, 105 respondents (99%) indicated that the majority of patients is satisfied with the functional results at 1 year of nonoperative treatment. Of these, 94 (89%) respondents indicated that 75–100% of the patients are satisfied with their functional outcome. Fewer respondents indicated similar results for Rockwood III ACJ injuries (*n* = 62, 85%). Concerning cosmetic results, 66 (62%) respondents indicated that 75–100% of the patients with a Rockwood II ACJ injury are satisfied at 1 year of nonoperative treatment. Fewer respondents indicated the same satisfaction for Rockwood III ACJ injuries (*n* = 16; 15%).

**Fig. 8:**
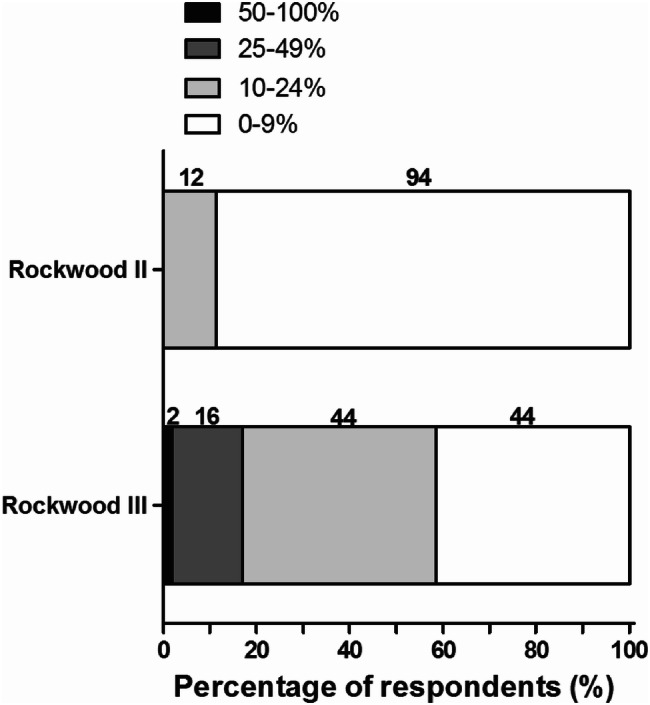
Respondents’ opinions on the proportion of patients that require secondary operative treatment after failed nonoperative management of a Rockwood II or III of ACJ injury. The number of respondents is given above the bars

Figure 8 shows the respondent’s opinion on the proportion of patients that require secondary operative treatment after failed nonoperative management of a Rockwood II or III ACJ injury. All respondents and 88 (83%) respondents indicated that less than 25% of patients need secondary operative treatment for Rockwood II or III ACJ injuries, respectively.

**Fig. 9:**
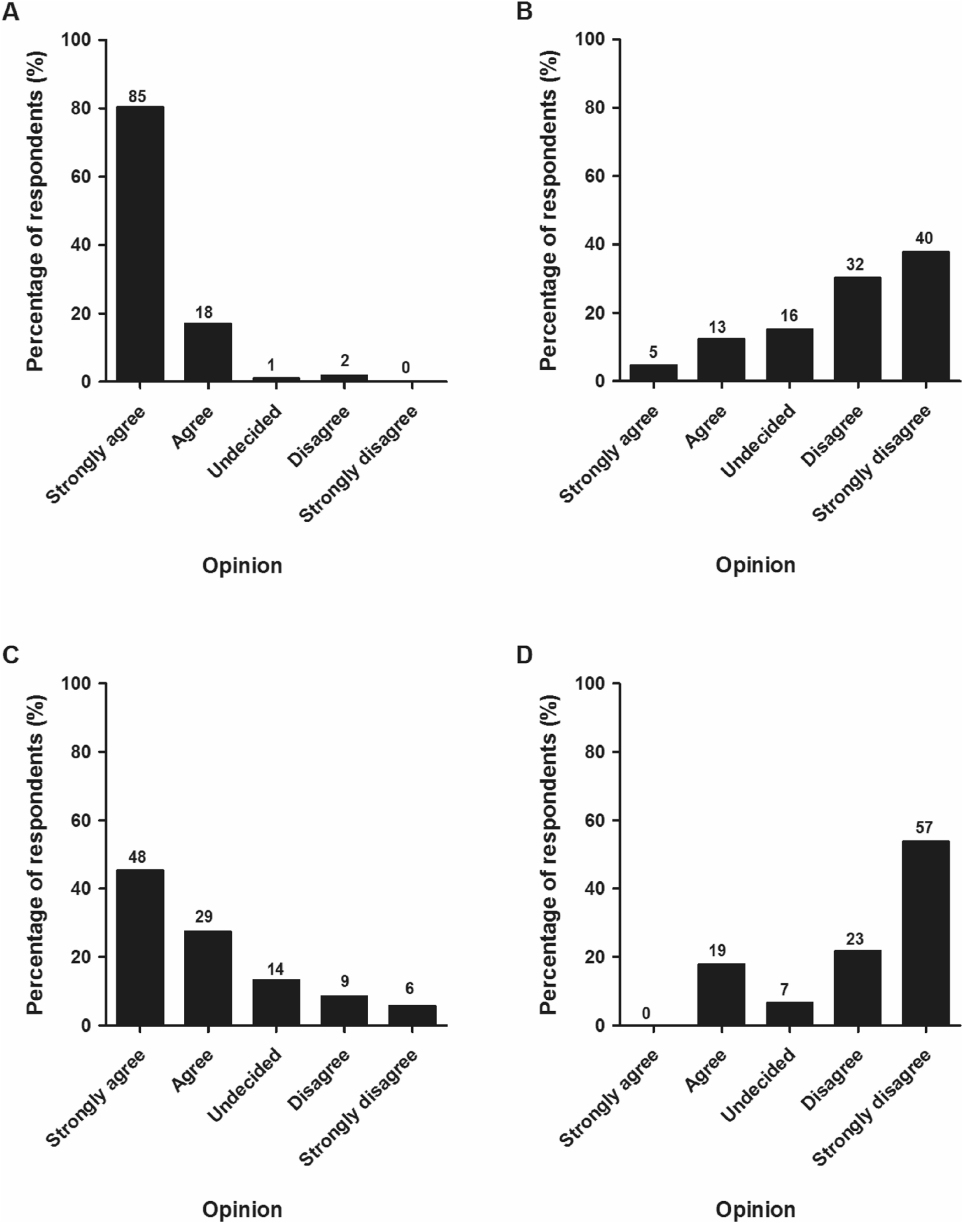
Respondents’ opinions regarding four statements: Statement A: “Primary surgical treatment is not indicated for a Rockwood II ACJ injury”; Statement B: “Healthy active patients with a Rockwood III ACJ injury should primarily be treated operatively”; Statement C: “Healthy active patients with a Rockwood IV ACJ injury should primarily be treated operatively”; Statement D: “With current surgical techniques, cosmetic complaints of an ACJ injury with a good shoulder function, should also be an indication for operative treatment”. The number of respondents is given above the bars

Figure 9 shows whether the respondents agree or disagree with different statements on the treatment of ACJ injuries. On the statement “There is no indication for primary operative treatment of a Rockwood II ACJ-dislocation”, 103 (97%) of the respondents (strongly) agreed. Also the majority (*n* = 72; 68%) respondents (strongly) disagreed to the statement that “Healthy and active patients with a Rockwood III ACJ injury should usually be treated operatively”.

On the statement “Healthy active patients with a Rockwood IV ACJ injury should usually be treated operatively, 77 (73%) respondents (strongly) agreed. On the statement “With current surgical techniques, cosmetic complaints of an ACJ injury with a good shoulder function should also be an indication for operative treatment”, 80 (76%) (strongly) disagreed.

## Discussion

Rockwood II and III were the most frequently seen ACJ injuries in The Netherlands. The diagnosis was mainly based on physical examination (91%) and radiographs (95%). The Rockwood classification was used the most. Rockwood II and III ACJ injuries were usually treated non-operatively, whereas Rockwood IV and V ACJ injuries were usually treated operatively. Patient factors contributing to the decision for operative treatment were mainly functional need and age. A flexible implant (e.g., Lockdown™) was preferred for primary and secondary operative treatment. Distal clavicle resection was more often deemed necessary during secondary than during primary operative treatment. All respondents agreed that nonoperative treatment of Rockwood II ACJ injuries leads to satisfactory results and that just rarely secondary operative treatment is required. The percentage of Rockwood III ACJ injuries requiring a secondary surgical intervention seemed higher, and the expected cosmetic result of non-operatively treated Rockwood III ACJ injuries was reported as less satisfactory. Hootman reported in a follow-up study of a previous review, that the evidence does not support (immediate) surgical treatment of grade III AC dislocations with respect to overall patient satisfaction as well as clinical outcomes such as pain, range of motion, and strength [13, 14]. Similar as in the current study, over 80% of patients with an ACJ type III injury treated non-operatively achieved satisfactory functional results and did not require surgery. Also, the Canadian Orthopaedic Trauma Society showed in a prospective randomized clinical trial good or excellent long term results of non-operative treatment of Rockwood III ACJ injuries, however, compared to Hook plate fixation [15].

Literature reports Rockwood II, III, and V to be the most common types of ACJ injuries [3].

It is unclear why the respondents in the current study report a lower rate of occurrence of Rockwood V ACJ injuries. The diagnostic approach reported by the respondents is in line with the literature. A systematic review showed high inter- and intra-observer reliability for diagnosing vertical instabilities of the clavicle using X-ray alone. Reliability for horizontal instabilities is much more variable [16].

Functional need (88%) and age (86%) were the most important patient-related factors to decide for operative management. In line with current general opinion, cosmesis (18%) seemed not to play a significant role in this decision. However, the cosmetic consequence of a completely displaced ACJ joint is hard to ignore. The respondents indeed expected patients with Rockwood III ACJ injuries to be less satisfied with cosmetic results. In a recent review, Chang et al. reported a poor cosmesis in both operative and nonoperative patients [8]. Hypertrophic or prominent scars were the most common complaint in the operative group, whereas deformity of the shoulder was more prevalent in the nonoperative group. Overall, cosmetic outcomes favored the operative group. Chang et al. emphasized that the idea of a poor cosmetic result is individual and subjective and does not necessarily correlate with the reduction of the AC joint [8].

Surprisingly, approximately a third of the respondents would not treat Rockwood IV and V ACJ dislocations primarily operatively. This is in contradiction with the general recommendation for primary operative treatment of Rockwood type IV to VI lesions, although the level of evidence of this recommendation is low [17–19]. A recent study found no correlation between Rockwood grade and clinical symptoms, suggesting that the reliability of using the Rockwood grade as a decision-making tool in the management of acute AC joint dislocation is unclear [20].

Whereas operative treatment is generally advised for Rockwood V ACJ injuries, the treatment of Rockwood III ACJ dislocations is still debated. Sixty-three (59%) respondents would treat a Rockwood III different than a Rockwood V ACJ injury, but only nine reported to base this distinction on additional radio-diagnostic tests. The data do not provide further details as to how they did make the distinction. In a small case study, it was found that MRI can allow the good anatomical display of ACJ structures and can give clinically relevant information on type and extension of ACJ injury [21]. In our survey, only two respondents used MRI and one used ultrasound for this purpose.

Previous studies have reported physiological clavicle rotations up to 30 degrees during shoulder abduction, elevation, retraction, and backward rotation [22–26]. Flexible implants allow the clavicle to move in a more or less natural anatomical fashion relative to the scapula. The vast majority of respondents recognized this phenomenon and use these implants in both primary and secondary operative treatment of any ACJ injury. For secondary surgery, flexible implants were often combined with a Weaver–Dunn procedure [27]. The array of implants used in the Netherlands is in line with previous publications [5–7]. Boffano et al. concluded that there is no consensus on the implant use, but suggest to treat young patients with high-grade ACJ injuries in the early stages using synthetic devices with open or arthroscopic procedures aiming to obtain a stable joint [5].

Rigid coracoclavicular screw fixation appears to be abandoned in the Netherlands. Rigid fixation of the clavicle to the scapula is non-physiological and hinders normal shoulder function for the period that the implant is present. This implicates that studies comparing flexible implants with nonoperative management might result more in favor of operative management than the available studies comparing rigid fixation techniques with nonoperative management.

Distal clavicle resection is more often considered necessary in secondary operative treatment. The reason for that might be shortening of the shoulder during nonoperative management of unstable ACJ injuries, hence the strut-function of the clavicle is lost after complete ACJ dislocation. Hillen et al. showed in a cadaveric study that clavicle shortening results in changes of maximal muscle moments around the shoulder girdle [28]. Nonoperative management of an unstable ACJ injury could have the same effect, resulting in shortening of muscles of the shoulder and inability to reduce the AC joint without too much tension. However, no high-quality clinical studies are present in this hypothesis.

With only two respondents considering to refer their patient to a colleague for expertise, there seems no need for centralization of this topic in The Netherlands.

A limitation of the current study is that, as is inherent to questionnaires, the level of scientific evidence is low.

## Conclusions

On many topics concerning diagnosis and treatment of ACJ-dislocations this survey showed a gross individual variation of strategies between (orthopedic) trauma surgeons in the Netherlands. Also in the available international literature, no clear guidelines are formulated yet. Many different operative treatment strategies have been compared with nonoperative management while, in the meantime, technical development of modern devices lead to new operation techniques. To the opinion of the majority of the Dutch surgeons, a flexible implant is the most promising technique at this moment. Further research is definitely needed to explore the functional, cosmetic, and economical results of nonoperative versus operative treatment with modern implants in complete unstable ACJ injuries. Ideally, such future studies are prospective and randomized.

## Electronic supplementary material

Below is the link to the electronic supplementary material.

Supplementary material 1 (DOCX 456 kb)
