# Supplementary material for: Current practice in the management of acromioclavicular joint dislocations; a national survey in the Netherlands
Source: Eur J Trauma Emerg Surg. 2020 Jun 13;47(5):1417–27. doi: 10.1007/s00068-020-01414-0 (PMC8476372; doi:10.1007/s00068-020-01414-0)
Supplement: Supplementary file 1 — Supplementary material 1 (DOCX 456 kb) [file 68_2020_1414_MOESM1_ESM.docx]

**National survey treatment acromioclavicular joint (ACJ) dislocations:**

1a) What is your age?

1b) What is your sex? [male /female]

1c) In what kind of hospital do you work most of the time?

- General
- High-volume non-academic trauma center
- High volume academic trauma center

1c) What is your specialty?

- Traumasurgeon
- Orthopedic surgeon
- Others [free tekst box]

1d) How many years do you work as a consultant traumasurgeon / orthopedic surgeon?

- 0 – 4 years
- 5 – 9 years
- 10 – 19 years
- > 20 years

2) How many percent of your total patient population consists of trauma patients?

- 0-19%
- 20-49%
- 50-74%
- 75-100%

3a) Estimate how many patients with an acute* ACJ dislocation does your surgeon group treat each year, non-operatively as well as operatively?

* Acute is defined as onset of treatment within two weeks after injury.

- 0-4
- 5-9
- 10-19
- 20-49
- >= 50

3b) Estimate what percentage of these patients is treated operatively in the acute phase*?

* Acute is defined as operative treatment within two weeks after injury

- 0-24%
- 25-49%
- 50-74%
- 75-100%

4) In literature, the most used classifications for ACJ dislocations are the Tossy or Rockwood classification. Rockwood I, II en III are similar to Tossy I, II en III.

[
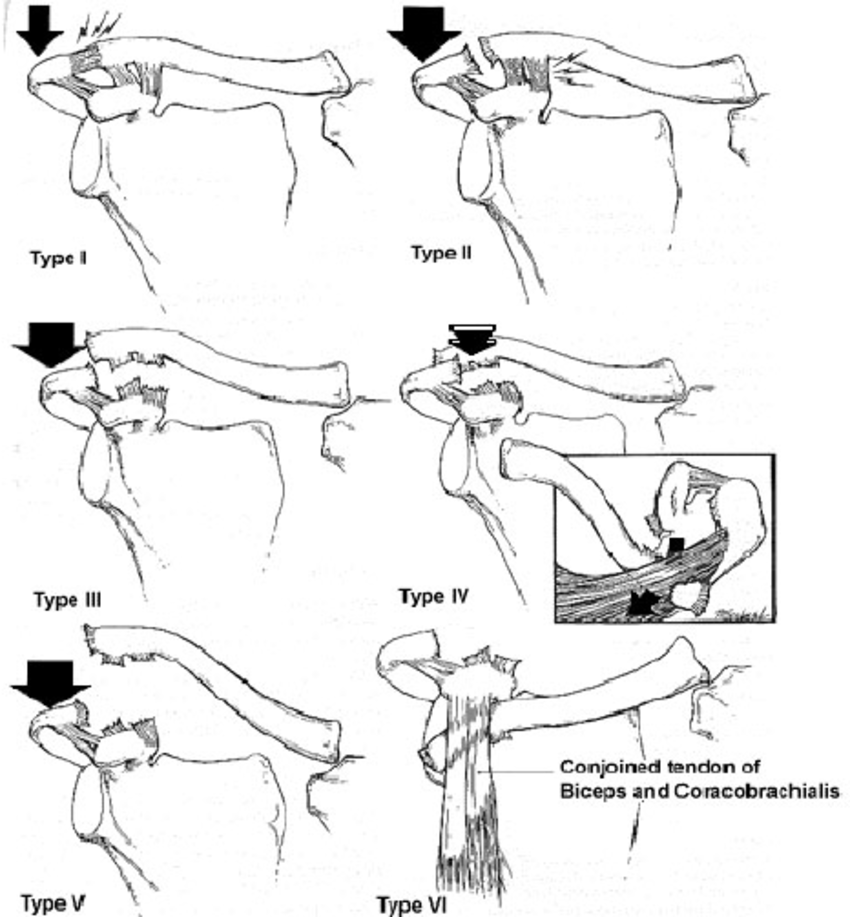
](https://www.google.nl/url?sa=i&rct=j&q=&esrc=s&source=images&cd=&cad=rja&uact=8&ved=0ahUKEwjjkOOSiKrNAhXC6RQKHb0xCJoQjRwIBw&url=https://www.researchgate.net/figure/33957962_fig2_Fig-2-Rockwood-Classification-of-acromioclavicular-joint-injury&bvm=bv.124272578,d.ZGg&psig=AFQjCNGtb3vKqaAThQTYS5Rqwg6gqRYxPg&ust=14660811789560)

4a) Which classification do you prefer to use in your clinical practice?

- Tossy
- Rockwood
- Other classification, namely…..[free text box]
- I do not use any classification (proceed to question 5)

4b) Based on which diagnostic methods do you classify the severity of an ACJ dislocation? (More options possible, please mark which is applicable)

- Physical examination: piano key phenomenon
- Physical examination: anteroposterior (horizontal) instability
- X-ray: Standard AP and lateral shoulder radiograph
- X-ray: Zanca view (10° cranial tilt)
- X-ray: Weight-bearing and non-weight bearing radiograph
- Ultrasound
- MRI
- Others, …..[free tekst box]

5) Do you have a protocol for treatment of acute ACJ dislocations in your surgeon group?

- Yes
- No
- I don’t know

6) In which matter do the factors shown below contribute to your decision to operative treatment of an acute ACJ dislocation?

6a) General factors:

- Hospital protocol [ never – hardly – neutral – mostly – always]
- Literature [ never – hardly – neutral – mostly – always]
- Congress / course [ never – hardly – neutral – mostly – always]
- Own experience [ never – hardly – neutral – mostly – always]

6b) Patient factors:

- Patient age [ never – hardly – neutral – mostly – always]
- Patient sex [ never – hardly – neutral – mostly – always]
- Functional need [ never – hardly – neutral – mostly – always]
- Cosmesis [ never – hardly – neutral – mostly – always]
- Patient preferences [ never – hardly – neutral – mostly – always]

7a) Estimate how many patients with an acute* ACJ dislocation are treated by your surgeon group each year (both non-operatively and operatively).

* Acute is defined as onset of treatment within two weeks after injury

- 0-4
- 5-9
- 10-19
- 20-49
- >= 50

7b) Your surgeon group treats an otherwise healthy and vital patient with an acute Tossy II (Rockwood II) ACJ dislocation:

- - (In principle) Non-operatively
  - (In principle) Operatively
  - Only operatively after failure of nonoperative management (after > three months)
  - Never operatively
  - Others, … [Free tekst box]

8a) Estimate how many patients with an acute* Tossy III (Rockwood III) ACJ dislocation are treated by your surgeon group each year (both non-operatively and operatively).

* Acute is defined as onset of treatment within two weeks after injury

- 0-4
- 5-9
- 10-19
- 20-49
- >= 50

8b) Your surgeon group treats an otherwise healthy and vital patient with an acute Tossy III (Rockwood III) ACJ dislocation:

- - (In principle) Non-operatively
  - (In principle) Operatively
  - Only operatively after failure of nonoperative management (after > three months)
  - Never operatively
  - Others, … [Free tekst box]

9) Rockwood differentiates between type III and type V ACJ dislocations. Type III is characterized by a rupture of both the AC and the CC ligaments. A type V is characterized by a rupture of both the AC and the CC ligaments, but also an avulsion of the deltoid fascia, resulting in more (>100%) elevation of the distal clavicle opposite to the acromion.

[
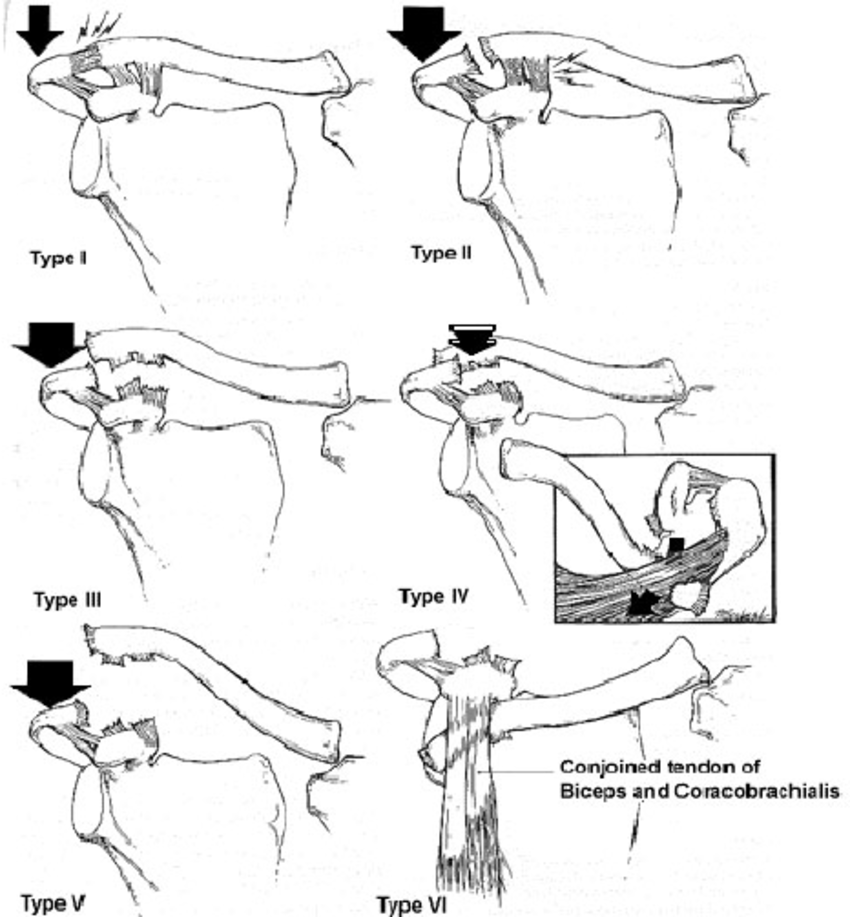
](https://www.google.nl/url?sa=i&rct=j&q=&esrc=s&source=images&cd=&cad=rja&uact=8&ved=0ahUKEwjjkOOSiKrNAhXC6RQKHb0xCJoQjRwIBw&url=https://www.researchgate.net/figure/33957962_fig2_Fig-2-Rockwood-Classification-of-acromioclavicular-joint-injury&bvm=bv.124272578,d.ZGg&psig=AFQjCNGtb3vKqaAThQTYS5Rqwg6gqRYxPg&ust=14660811789560)

9a) Do you deliberately make a distinction in your diagnostic process between a Rockwood type III and type V ACJ dislocation?

- Yes
- No

9b) Do you use additional diagnostic tools in order to differentiate between a Rockwood type III and type V ACJ dislocation in your patients?

- No
- Yes, I use
- X-ray: Weight bearing (weight on injured side)
- Ultrasound
- MRI
- Others, …..[free tekst box]

9c) Do you make a distinction between Rockwood type III and type V ACJ dislocations concerning your choice of treatment?

- Yes [proceed to 9d]
- No

9d) Your surgeon group treats an otherwise healthy and vital patient with an acute Rockwood type V ACJ dislocation:

- - (In principle) Non-operatively
  - (In principle) Operatively
  - Only operatively after failure of nonoperative management (after > three months)
  - Never operatively
  - Others, … [Free tekst box]

10a) If you treat an acute ACJ dislocation (within twee weeks after injury) operatively, which operative technique do you prefer? Multiple options allowed, please select a maximum of three:

- - - K-wire fixation / Zuggurtung cerclage
    - Clavicula hook-plate
    - (Modified) Weaver-Dunn procedure
    - Bosworth-screw (coracoclavicular screw fixation)
    - Coracoclavicular suture / tape / button suture (select any applicable)
      - - Coracoclavicular suture (vicryl / mersilene or comparable)
- Coracoclavicular tape (Vicryl / mersilene or comparable)
- Coracoclavicular button-suture (Tightrope, Arthrex)
- Coracoclavicular button-suture (AC Dogbone, Arthrex)
- Coracoclavicular tape (Lockdown^TM^, Oudshoorn)
- Coracoclavicular suture / tape / button suture combined with Weaver-Dunn procedure
  - - Others, ….. [Free tekst box]

10b) How often do you consider a distal clavicle resection necessary during acute operative treatment of an ACJ dislocation?

- Always
- Mostly
- Rarely
- Never

10c) If you treat an ACJ dislocation operatively after initial non-operative treatment (more than three months after injury), which operative technique do you prefer? Multiple options allowed, please select a maximum of three:

- - - K-wire fixation / Zuggurtung cerclage
    - Clavicula hook-plate
    - (Modified) Weaver-Dunn procedure
    - Bosworth-screw (coracoclavicular screw fixation)
    - Coracoclavicular suture / tape / button suture (select any applicable)
      - - Coracoclavicular suture (vicryl / mersilene or comparable)
- Coracoclavicular tape (Vicryl / mersilene or comparable)
- Coracoclavicular button-suture (Tightrope, Arthrex)
- Coracoclavicular button-suture (AC Dogbone, Arthrex)
- Coracoclavicular tape (Lockdown^TM^, Oudshoorn)
- Coracoclavicular suture / tape / button suture combined with Weaver-Dunn procedure
- Others, ….. [Free tekst box]

10d) How often do you consider a distal clavicle resection necessary during such delayed (> three months after injury) operative treatment of an ACJ dislocation?

- Always
- Mostly
- Rarely
- Never

11a) What is in your opinion the percentage of patients with a Tossy II (Rockwood II) ACJ dislocation that is satisfied with the functional results after one year of non-operative management?

- 0-24%
- 25-49%
- 50-74%
- 75-100%
- I don’t know

11b) What is in your opinion the percentage of patients with a Tossy II (Rockwood II) ACJ dislocation that is satisfied with the cosmetic results after one year of non-operative management?

- 0-24%
- 25-49%
- 50-74%
- 75-100%
- I don’t know

12a) What is in your opinion the percentage of patients with a Tossy III (Rockwood III) ACJ dislocation that is satisfied with the functional results after one year of non-operative management?

- 0-24%
- 25-49%
- 50-74%
- 75-100%
- I don’t know

12b) What is in your opinion the percentage of patients with a Tossy III (Rockwood III) ACJ dislocation that is satisfied with the cosmetic results after one year of non-operative management?

- 0-24%
- 25-49%
- 50-74%
- 75-100%
- I don’t know

13a) What is in your opinion the percentage of patients with a Tossy II (Rockwood II) ACJ dislocation that, after initial nonoperative management, still require secondary operative treatment?

- 0-9%
- 10-24%
- 25-49%
- 50-100%

13b) What is in your opinion the percentage of patients with a Tossy III (Rockwood III) ACJ dislocation that, after initial nonoperative management, still require secondary operative treatment?

- 0-9%
- 10-24%
- 25-49%
- 50-100%

14a) Estimate how many patients with an acute* Rockwood IV ACJ dislocation are treated by your surgeon group each year (both non-operatively and operatively)?

- - 0-1
  - 2-4
  - 5-9
  - 10 or more

14b) Your surgeon group treats an otherwise healthy and vital patient with an acute Rockwood IV ACJ dislocation:

- - (usually) Non-operatively
  - (usually) Operatively
  - Only operatively after failure of nonoperative management (after > three months)
  - Never operatively

15) Thesis: “There is no indication for primary operative treatment of a Tossy II (Rockwood II) ACJ-dislocation”

- Strongly agree
- Agree
- Undecided
- Disagree
- Strongly disagree

16) Thesis: “Healthy and active patients with a Tossy III (Rockwood III) ACJ dislocation should usually be treated operatively”.

- Strongly agree
- Agree
- Undecided
- Disagree
- Strongly disagree

17) Thesis: “Healthy and active patients with a Rockwood IV ACJ dislocation should usually be treated operatively”.

- Strongly agree
- Agree
- Undecided
- Disagree
- Strongly disagree

18) Thesis: “With current operation techniques, cosmetic complaints of an ACJ injury with a good shoulder function, should also be an indication for operative treatment.”

- Strongly agree
- Agree
- Undecided
- Disagree
- Strongly disagree
